# The Tec Kinase Itk Integrates Naïve T Cell Migration and *In Vivo* Homeostasis

**DOI:** 10.3389/fimmu.2021.716405

**Published:** 2021-09-09

**Authors:** Flavian Thelen, Stefanie Wissmann, Nora Ruef, Jens V. Stein

**Affiliations:** ^1^Department of Medical Oncology and Hematology, University of Zürich and University Hospital Zürich, Zürich, Switzerland; ^2^Department of Oncology, Microbiology and Immunology, University of Fribourg, Fribourg, Switzerland

**Keywords:** T cell trafficking, chemokine, signal transduction, Tec kinase, CCL21/CCR7 axis, intravital 2-photon microscopy

## Abstract

Naïve T cells (T_N_) constitutively recirculate through secondary lymphatic organs (SLOs), where they scan dendritic cells (DCs) for cognate peptide-loaded major histocompatibility complexes (pMHC). Continuous trafficking between SLOs not only enables rapid clonal selection but also ensures T_N_ homeostasis by providing access to prosurvival signals from TCR, IL-7R, and the chemokine receptor CCR7. Inside the lymphoid tissue, CCR7-mediated T_N_ motility is mainly driven by the Rac activator DOCK2, with a separate contribution by a phosphoinositide-3-kinase γ (PI3Kγ)-dependent pathway. Tec tyrosine kinases and the Rac activator Tiam1 constitute prominent downstream effectors of PI3K signaling. Yet, the precise role of Tec kinase versus Tiam1 signaling during CCR7-mediated T_N_ migration and homeostasis remains incompletely understood. Here, we examined the function of the Tec family member interleukin-2-inducible T-cell kinase (Itk) and Tiam1 during T_N_ migration *in vitro* and *in vivo* using intravital microscopy. Itk deficiency caused a mild decrease in CCR7-triggered T_N_ migration, mirroring observations made with PI3Kγ;^-/-^ T cells, while lack of Tiam1 did not affect T_N_ motility. *In silico* modeling suggested that reduced migration in the absence of Itk does not result in a substantial decrease in the frequency of T_N_ encounters with DCs within the lymphoid tissue. In contrast, Itk was important to maintain *in vivo* homeostasis of CD4^+^ T_N_, also in MHCII-deficient hosts. Taken together, our data suggest that Itk contributes to T_N_ migration and survival by integrating chemokine receptor and TCR signaling pathways.

## Introduction

Naïve T cells (T_N_) continuously roam secondary lymphoid organs (SLOs) including peripheral lymph nodes (PLNs) to scan for the presence of cognate peptide-loaded major histocompatibility complex (pMHC) on dendritic cells (DCs). The chemokine receptor CCR7 on T_N_ and its ligands CCL19 and CCL21 expressed by SLO stromal cells fulfill a key function in this process (1). CCL21 is central for recruitment of blood-borne T_N_ *via* high endothelial venules into the PLN parenchyma (2), where CCR7 ligands contribute to fast T_N_ motility of ~15 µm/min on a scaffold of fibroblastic reticular cells (FRCs) (3–7). This process enables efficient T_N_ scanning of pMHC presented on DC surfaces (8, 9). In addition to providing an “antigen library” that mirrors the immune status of their surveilled area, SLOs provide critical factors for T_N_ homeostasis. First, FRCs are a main source of the prosurvival cytokine IL-7 (10). Second, tonic signaling by self-pMHC binding to the T cell receptor (TCR) on migrating T cells induces a baseline phosphorylation of the TCR complex ζ; chain important for responsiveness to foreign pMHC (11) and CD4^+^ T cell survival (12, 13). Third, CCR7 ligands themselves constitute prosurvival signals for circulating T_N_ (10). Accordingly, the expression of CCR7, the PLN homing receptor CD62L, and the IL-7 receptor CD127 are transcriptionally coordinated in T_N_ (14).

On a molecular level, CCR7 activation triggers an increase in F-actin polymerization, mainly *via* the Rac guanine exchange factor (GEF) DOCK2 that catalyzes the formation of Rac-GTP at the cell’s leading edge. This, in turn, activates the actin nucleator Arp2/3 complex *via* the nucleation promoting factor Scar/WAVE (15, 16). DOCK2-Rac-Scar/WAVE-Arp2/3-driven retrograde (i.e., from the cell front to the rear) F-actin flow imprints the characteristic amoeboid shape of migrating T_N_ and forms the basis for rapid cellular translocation by force-coupling of cortical actin to the extracellular substrate *via* transmembrane receptors, mainly LFA-1 (17).

In addition to DOCK2 activation, chemokine receptors activate a phosphoinositide-3-kinase (PI3K) pathway in T cells (18). In T_N_, CCR7 signaling activates the p110γ isoform through its interaction with Gβγ subunits that dissociate from Gα_i_ after ligand binding (19). While pharmacological or genetic inhibition of this pathway does not affect the major DOCK2-Rac-Arp2/3 axis, PI3K blockade induces a minor but significant decrease in T_N_ migration *in vitro* and *in vivo* (19, 20). To date, the downstream signals that transmit PI3K signals for CCR7-mediated T_N_ migration remain incompletely understood. PI3K-generated phosphoinositide-3,4,5-triphosphate (PIP3) at the inner plasma membrane is recognized by proteins containing pleckstrin homology (PH) domains, such as the Rac GEF Tiam1, which has been implicated in T cell trafficking (21, 22). Additionally, Tec proteins constitute a well-characterized PH-domain-containing nonreceptor tyrosine kinase family that plays key regulatory functions in lymphocyte development, activation, and effector differentiation. The Tec family is composed of five members, of which Itk and Rlk are expressed in T_N_ (23). Itk signals from multiple surface receptors, such as TCR, the costimulatory receptor CD28, and chemokine receptors (23–25). In accordance with this, Itk-deficient and Itk/Rlk double-deficient T cells show reduced chemotaxis to CXCL12 (26, 27). Itk activation of Vav1, a GEF for the small GTPases Rac1 and Cdc42, is important for actin reorganization and adhesion (23, 28–30). Yet, the precise roles of Itk and Tiam1 during CCR7-triggered DOCK2- versus PI3K-dependent T_N_ migration and their impact on physiological T_N_ motility and homeostasis have not been assigned thus far.

Here, we investigated the role of Itk and Tiam1 in CCR7-driven T_N_ motility and survival after adoptive transfer. Our data uncover a role for Itk but not Tiam1 downstream PI3K-dependent *in vitro* T_N_ polarization and migration in response to the CCR7 ligand CCL21, with a concomitant decrease in homing capacity to SLOs. While intravital imaging confirmed that *in vivo* T cell motility is reduced in the absence of Itk, *in silico* track modeling suggests only a minor impact on the efficacy to encounter DCs in the LN parenchyma. In contrast, significantly fewer Itk-deficient CD4^+^ T cells were recovered from blood and SLOs 8–14 days after adoptive transfer into WT and MHCII-deficient recipients, supporting a role for this Tec family member for T_N_ homeostasis even in the absence of tonic TCR signaling.

## Results

### CCL21-Induced T Cell Polarization and Migration Are Impaired in the Absence of Itk

**Figure 1:**
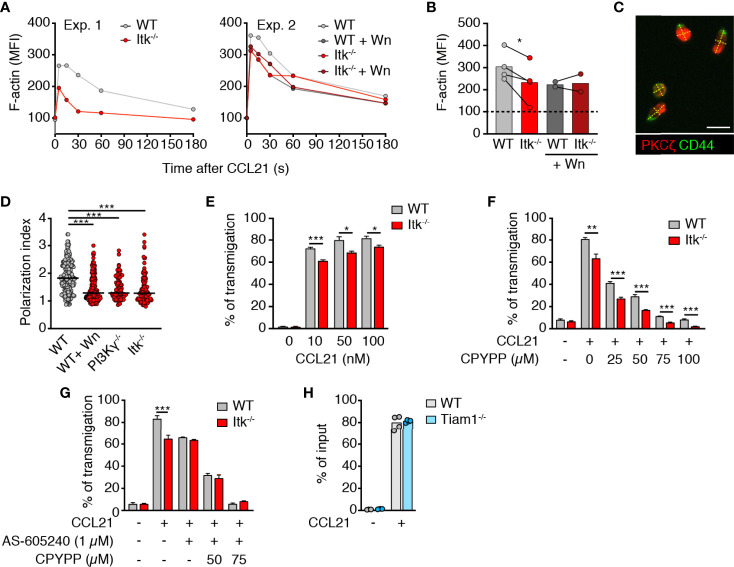
Itk enhances CCL21-induced polarization and migration of T cells. **(A)** MFI of F-actin after CCL21 stimulation with and without Wn treatment. **(B)** Summary of normalized F-actin MFI at 30 s post CCL21 addition (100 = baseline). Experiments performed on the same day (i.e., same flow cytometer settings) are linked with a line. **(C)** Image of CCL21-stimulated T cells on fibronectin-coated glass dish. Scale bar, 10 µm. **(D)** Quantification of cell polarization as measured by the ratio between the length and the width of the cellular body (dotted yellow lines in C). **(E)** Chemotaxis of WT and Itk^-/-^ CD4^+^ T_N_ to indicated CCL21 concentrations. **(F, G)** Chemotaxis of WT and Itk^-/-^ CD4^+^ T_N_ pretreated with CPYPP **(F)** and/or AS-605204 **(G)** toward 50 nM CCL21. Graph shows percentage of transmigrated cells. **(H)** Chemotaxis of WT and Tiam1^-/-^ CD4^+^ T cells toward 50 nM CCL21. Graph shows percentage of transmigrated cells. Data in **(B)** were analyzed using a paired t-test. Data in **(D–H)** are pooled from at least two independent experiments and analyzed by Kruskal–Wallis test **(D)** or unpaired Student’s t-test **(E–H)**. *p < 0.05; **p < 0.01; ***p < 0.001.

We first examined the impact of Itk deficiency on CCR7-triggered rapid F-actin polymerization. Akin to PI3Kγ^-/-^ T cells (19), we detected a small but consistent reduction of F-actin polymerization in Itk^-/-^ T cells immediately after CCL21 addition (**Figures 1A, B**). This difference disappeared when WT cells were pretreated with Wortmannin (Wn), a broad PI3K inhibitor, whereas the same treatment on Itk^-/-^ cells caused no further decrease in F-actin polymerization (**Figures 1A, B**). This is in line with results showing that Itk acts downstream of PI3K and contributes to F-actin polymerization upon chemokine receptor activation in T cells (23). Given the close causal relation between F-actin treadmilling and T cell shape (17), we examined the impact of Itk on chemokine-induced polarization. T cells were allowed to adhere to fibronectin-coated glass slides and stimulated with CCL21 for 20 min, followed by immunofluorescent analysis of PKCζ and CD44 as markers for leading edge and uropod, respectively (**Figure 1C**). Itk^-/-^ T cells showed reduced cellular polarization as compared to WT T cells and resembled PI3Kγ^-/-^ T cells or WT T cells treated with the pan-PI3K inhibitor Wortmannin (Wn) (**Figure 1D**). Taken together, our data suggest that a PI3K-Itk pathway contributes to F-actin polymerization and T cell polarization upon stimulation with CCR7 ligands.

Itk^-/-^ mice display a defect in thymic positive selection and show an increased ratio of “memory-like” CD44^high^ to CD44^low^ T_N_ in SLOs (31, 32) (**Figure S1A**). To exclude this as cause of reduced Itk^-/-^ T cell response to CCL21, we enriched CD62L^high^CD44^low^ *bona fide* T_N_ by depleting CD44^high^ T cells using titrated amounts of anti-CD44 mAb-coated magnetic beads as described (33) and applied bead sorting to both WT and Itk^-/-^ T cells (**Figure S1B**). As reported for migration to CXCL12 (26, 27), *bona fide* Itk^-/-^ T_N_ migration toward increasing concentrations of CCL21 showed a minor but significant and consistent reduction (**Figure 1E**).

In contrast to Itk, DOCK2 activity in lymphocytes is regulated in a PI3K-independent manner (19) and plays a pivotal role in chemokine-induced migration (15). Accordingly, we observed reduced migration of both WT and Itk^-/-^ T_N_ with the specific DOCK2 inhibitor CPYPP (34) in a dose-dependent manner (**Figure 1F**). The migratory difference between WT and Itk^-/-^ CD4^+^ T cells was maintained during CPYPP treatment, confirming that Itk-dependent migration is uncoupled from DOCK2-induced motility. In line with this, treatment of WT CD4^+^ T_N_ with the specific PI3Kγ inhibitor AS-605240 reduced migration to the same level as of Itk^-/-^ CD4^+^ T_N_, while this treatment did not have an impact on Itk^-/-^ CD4^+^ T_N_ chemotaxis (**Figure 1G**). Consistent with the abolished chemotaxis of DOCK2 x PI3Kγ-double deficient T cells (19), combined CPYPP + AS-605240 treatment completely abrogated T_N_ migration to CCL21 (**Figure 1G**). Similarly, migration toward CXCL12 was partially reduced in WT T_N_ by either CPYPP or AS-605240 treatment, while combined CPYPP + AS-605240 treatment abolished T_N_ chemotaxis (**Figure S1C**). In contrast, Itk^-/-^ T_N_ migration to CXCL12 was not affected by AS-605240, whereas CPYPP treatment severely impaired chemotaxis toward CXCL12 (**Figure S1C**). These data suggested that the dual engagement of DOCK2- and PI3Kγ-dependent signaling affects other chemokine receptors as well. Inhibitor treatment did not affect the viability of T_N_, as assessed by PI staining (>90% viability after double inhibitor treatment).

Itk has been reported to be constitutively associated with the Rac/Cdc42 GEF Vav1, which mediates its effects on the actin cytoskeleton (30). In addition to Vav1, the Rac GEF Tiam1 becomes activated downstream of PI3K signaling (35), and Tiam1 has been previously shown to mediate T cell polarization and migration to CXCL12 and CCL21 *via* the Par complex (22, 36). We therefore examined whether Tiam1^-/-^ T cells were impaired in their motility to CCL21. However, Tiam1^-/-^ CD4^+^ T cells showed no defect in chemotaxis (**Figure 1H**). Taken together, these results confirm that DOCK2 plays a dominant function in CCL21-dependent T_N_ migration, while a PI3Kγ-Itk axis contributes to optimal cell polarity and chemokine responsiveness, potentially by regulating the activity of Vav1 or other GEFs.

### Itk Supports T_N_ Migration in LN Parenchyma

**Figure 2:**
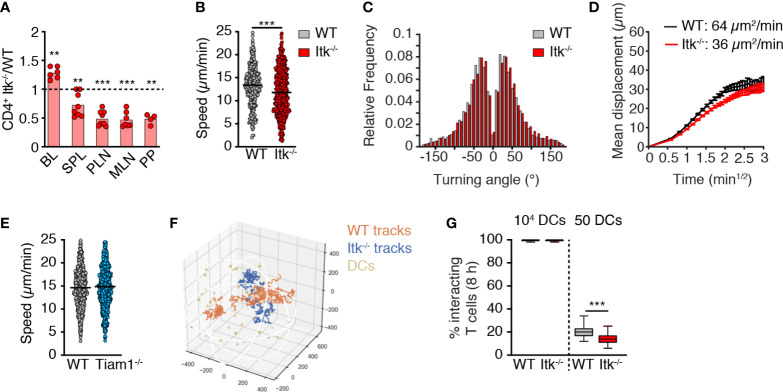
Itk contributes to *in vivo* T_N_ homing and motility. **(A)** Ratio of Itk^-/-^ to WT CD4^+^ T_N_ in blood (BL), spleen (SPL), PLN, mesenteric LN (MLN), and Peyer’s patches (PP) at 2 h post adoptive transfer. **(B, C)** Speeds **(B)** and frequency of turning angles **(C)** of WT and Itk^-/-^ CD4^+^ T_N_ in LN parenchyma. **(D)** Mean displacement of T cells as a function of the square root of time. **(E)** Speeds of WT and Tiam1^-/-^ T_N_ in LN parenchyma. **(F)** Representative 8-h-long *in silic*o WT and Itk^-/-^ CD4^+^ T cell tracks with 50 dispersed DCs. Numbers indicate µm. **(G)** Percentage of 100 *in silico* WT and Itk^-/-^ CD4^+^ T cell tracks encountering 1 in 10^4^ or 50 DCs in 50 8-h simulations. All data were pooled from at least two independent experiments (except PP in A; n = 4 mice from one exp.). Data in **(A)** were analyzed by a one-sample t-test against the theoretical value of “1” (= equal recovery); data in **(B, E, G)** were analyzed using an unpaired Student’s t-test, and data in **(C)** using Mann–Whitney test. **p < 0.01; ***p < 0.001.

Rlk/Itk-double-deficient naïve T cells display impaired *in vivo* homing to spleen and PLNs (27). We confirmed this observation after adoptive transfer of WT and Itk^-/-^ CD4^+^ T_N_, since we recovered fewer Itk-deficient T cells in spleen and other SLOs at 2 h post transfer, while these cells were more abundant in blood (**Figure 2A**). Since CCR7 contributes to T_N_ motility inside the LN parenchyma (3, 5, 6, 17, 37), we set out to assess the impact of Itk deficiency on *in vivo* T cell scanning behavior. We adoptively transferred fluorescently labeled CD4^+^ WT and Itk^-/-^ T_N_ into C57BL/6 recipients and performed intravital imaging of the popliteal LN 16-24 h post transfer. We detected a minor but significant reduction in Itk^-/-^ CD4^+^ versus WT T_N_ speeds (13.2 ± 0.3 and 11.9 ± 0.3 µm/min for WT and Itk^-/-^ T_N_, respectively; mean ± SEM; **Figure 2B**) without affecting the turning angles as a readout for directionality (38) (**Figure 2C**). As a result, the motility coefficient (MC), which represents a measure of the average scanned area per time, was reduced from 64 µm^2^/min for WT to 36 µm^2^/min for Itk^-/-^ CD4^+^ T_N_ (**Figure 2D**). In contrast, parenchymal CD4^+^ T cell speeds were unaffected by Tiam1 deficiency (**Figure 2E**), in line with *in vitro* chemotaxis data.

Based on the measured *in vivo* motility data, we modeled how T_N_ encounters with abundant (10^4^) or rare (50) DCs would be affected by Itk deficiency using *in silico* generated tracks in an artificial 3D volume representing the T cell zone (39) (**Figure 2F**). Both cell types efficiently intercepted highly abundant DCs (**Figure 2G**). In contrast to the reported reduction of T cell–DC encounters in the absence of DOCK2 (39), the absence of Itk only had a minor impact on the ability of migrating T_N_ to encounter rare cognate pMHC-bearing DCs (20.1 ± 4.9% and 13.8 ± 4.3% of WT and Itk^-/-^ *in silico* T cell tracks, respectively, engaging in DC encounters in 8-h simulations with a total of 50 DCs/T cell zone; mean ± SD; **Figure 2G**). Taken together, Itk signaling makes a minor but detectable contribution to *in vivo* T cell migration akin to observations made with PI3Kγ^-/-^ T cells (40), yet without causing a substantial decrease in the likelihood of T cell–DC encounters.

### Itk Contributes to Circulating T_N_ Homeostasis

**Figure 3:**
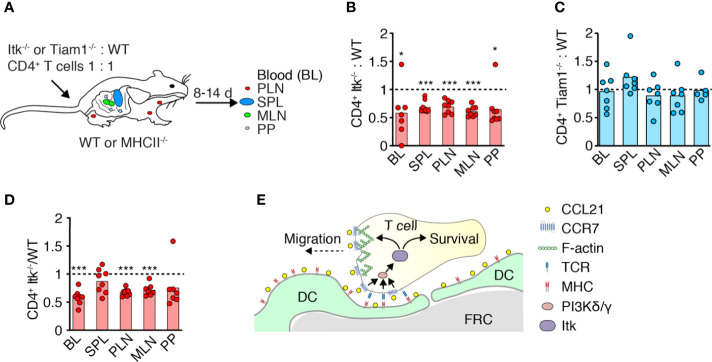
Itk supports physiological T_N_ homeostasis. **(A)** Experimental layout. Fluorescently labeled WT and Itk^-/-^ or Tiam1^-/-^ CD4^+^ T_N_ were transferred in a 1:1 ratio into WT or MHCII^-/-^ recipients, and blood (BL), spleen (SPL), PLN, mesenteric LN (MLN), and Peyer’s patches (PP) were analyzed 8–14 days post transfer (p.t.) by flow cytometry. **(B–D)** Itk^-/-^ or Tiam1^-/-^ to WT CD4^+^ T_N_ ratio recovered 10–14 days p.t. into WT **(B, C)** or 8 days p.t. into MHCII^-/-^ recipients **(D)**, normalized to input. **(E)** Scheme of Itk function in T_N_ migration and survival. Data in **(B–D)** are from two independent experiments and analyzed by a one-sample t-test against the theoretical value of “1” (= equal recovery). *p < 0.05; ***p < 0.001.

CD4^+^ T_N_ continuously homes to SLO to receive pro-survival factors provided in part by TCR and CCR7 signaling. We therefore investigated whether Itk promotes T_N_ homeostasis by facilitating homing and integration of prosurvival signals from pMHC and/or CCR7. We transferred fluorescently labeled WT and Itk^-/-^ CD4^+^ T_N_ at a 1:1 ratio into age- and sex-matched recipients as described (10). After 10–14 days, we isolated SLOs and blood to determine the ratio of recovered WT and Itk^-/-^ CD4^+^ T_N_ (**Figure 3A**). In all organs tested, we detected an approximately 30% reduction in the frequency of CD4^+^ T_N_ lacking Itk^-/-^ expression in comparison to their WT counterparts (**Figure 3B**). In contrast to the short-term homing experiment (**Figure 2A**), we now observed fewer Itk^-/-^ T_N_ in blood as compared to their WT counterparts. In contrast, we recovered a similar ratio of Tiam1^-/-^: WT T cells from all tissues at 14 days post transfer (**Figure 3C**).

To examine whether reduced recovery of Itk^-/-^ T cells is owing due to impaired homeostatic TCR signaling (24), we transferred Itk^-/-^and WT CD4^+^ T_N_ into MHCII^-/-^ recipients in a 1:1 ratio as above. On 8 days post transfer, we collected SLOs and measured the ratio of recovered WT: Itk^-/-^ CD4^+^ T cells. Similar to WT recipients, Itk^-/-^ CD4^+^ T_N_ were underrepresented in blood and most SLOs except spleen (**Figure 3D**). Taken together, these data suggest a role for Itk in mediating T_N_ homeostasis beyond TCR signal transduction, presumably by signal transmission *via* CCR7 or other chemoattractant receptors (**Figure 3E**).

## Discussion

T_N_ uses two separate signaling modules for CCR7-triggered motility, a major DOCK2-dependent and a minor PI3Kγ-dependent pathway (19, 40). While the DOCK2-Scar/WAVE-Arp2/3-Rac signaling cascade is well characterized, it has remained unclear which molecules act downstream PI3K after CCR7 activation. Here we report a role for the Tec kinase Itk in optimizing PI3K-dependent T_N_ polarization and migration. In line with the limited contribution of PI3K signaling for homeostatic T_N_ migration, we find that Itk deficiency had only a minor effect on CCR7-induced motility *in vitro* and T_N_ scanning behavior *in vivo*. In contrast, Itk deficiency impaired short-term T_N_ homing to SLOs and precipitated a loss of T_N_ recovery after 1–2 weeks post transfer. Thus, Itk integrates PI3K-dependent motility and homeostasis of circulating T_N_.

Unlike in neutrophils (41), DOCK2 activation in T cells occurs independently of PIP3 and might involve additional phospholipids such as phosphatidic acid (42). Given that PI3K activation is a universal hallmark of chemokine receptors including CCR7 (18), there is, to date, remarkably little knowledge on the physiological impact of this pathway in primary T cells. Tec kinases act at the crossroads of TCR and chemokine receptor signaling and have well-characterized effects on the actin cytoskeleton in multiple cell types, with a wide-ranging impact on T cell development, activation, and differentiation (23, 24). Here we focused on the role of Itk, since its activity is regulated by PIP3 binding to its PH domain, in contrast to the other Tec family member expressed in naïve T cells, Rlk (23) (**Figure S2**). While we identify a role for Itk in CCR7-triggered PI3K-dependent T_N_ migration, our findings suggest a more prominent role of Itk for long-term T_N_ survival. Our data further indicate that Itk-dependent homeostasis is not solely driven by TCR signaling, as evidenced by reduced recovery of Itk^-/-^ T_N_ in MHC II-deficient hosts as compared to WT T_N_. As TCR stimulates the PI3Kδ isoform in T cells, while CCR7 activates PI3Kγ, Itk is in a key position to integrate both baseline PIP3 signals generated during homeostatic T_N_ recirculation into a prosurvival program (**Figure 3E**). One caveat is that the I-Aa^-/-^ recipients we used as MHCII-deficient might still retain residual expression of noncanonical MHC molecules (12, 43).

In contrast to previous publications (22, 36), we were unable to assign a role for Tiam1 in CCR7-driven, PI3K-mediated T_N_ migration *in vitro* and *in vivo*. While the reason for this discrepancy might be due to the different mouse strain background used in both studies, our findings reflect the lack of Tiam1 expression in C57BL/6 T_N_ (www.immgen.org). Similarly, Tiam2 was not expressed in naïve CD4^+^ T cells (**Figure S2**). Furthermore, recent work suggests that the Tiam1 PH domain binds with a lower affinity to PIP3 as compared to other membrane lipids such as phosphoinositide-5-phosphate (44). Taken together, our data assign a role for Itk during CCR7-mediated signaling in T cells, leading to enhanced actin polymerization, polarization, and migration. Yet, the most relevant impact of Itk is in promoting T_N_ homeostasis, presumably by facilitating SLO homing and integration of prosurvival pMHC and CCR7 signals.

## Material and Methods

### Mouse Lines

C57BL/6 mice were purchased from Janvier labs. Itk^-/-^ (31) and Tiam1^-/-^ mice (45) were obtained from P. Schwartzberg (NIH, Bethesda, MD, USA) and J. van Buul (Sanquin Research Center, The Netherlands), respectively. PI3Kγ^-/-^ and H2-Aa^tm1Blt^ (MHCII^-/-^) were described (46, 47). Mice were bred at the Department of Clinical Research of the University of Bern and at the University of Fribourg. Experiments were approved by the Cantonal authorities and performed in accordance with the Swiss Federal Veterinary Office guidelines.

### T Cell Isolation

Spleens, PLNs, and mesenteric LNs (MLNs) were harvested and homogenized using a 70-µm cell strainer and a syringe plug. T cells or CD4^+^ T cells were isolated with an EasySep Mouse negative selection kit according to the manufacturer’s protocol (STEMCELL Technologies). For depletion of CD44^high^ CD4^+^ T cells, 2.5 µg of biotinylated anti-CD44 mAb (BD Biosciences) was added per 10^7^ cells. Cells were incubated for 20 min on ice. CD44^low^ *bona fide* T_N_ was negatively isolated using an EasySep™ Streptavidin RapidSpheres™ Isolation kit according to the manufacturer’s protocol (STEMCELL Technologies). Purity was typically >95%.

### Flow Cytometry

Single cell suspensions from SLOs were isolated and homogenized using 70-µm cell strainers. Fc receptors were blocked with purified anti-CD16/CD32 mAb (2.4G2) in a FACS buffer (PBS with 1% FCS and 0.05% NaN3) for 10 min. Cells were stained with fluorochrome-conjugated mAbs against CD8 (53-6.7), CD62L (Mel-14; 4°C for 30 min), CD4 (RM4-5; all Biolegend), and CD44 (IM7; BD Bioscience) with appropriate isotype controls and analyzed by flow cytometry (BD Biosciences). 

### F-Actin Polymerization, Cell Polarization, and Chemotaxis

For F-actin polymerization, T cells from Itk^-/-^, PI3Kγ^-/-^, and WT mice were starved for 1.5 h in a serum-free medium. Where noted, cells were pretreated with 0.5 µM Wortmannin (Calbiochem) for 1 h at 37°C. CCL21 (100 nM; Peprotech) was added to 2 x 10^6^ cells/ml in a 37°C water bath, and cells were fixed at indicated time points in 4% cold PFA. Cells were permeabilized and labeled with FITC-Phalloidin (Invitrogen) as described (19). For polarization assays, T cells were added on 1 µg/ml fibronectin-coated glass slides before the addition of CCL21 (100 nM) and fixation after 20 min with 2% PFA. After permeabilization with 0.1% Triton X-100 (5 min), cells were incubated with anti-protein kinase ζ; (PKCζ) (H-1; Santa Cruz Biotechnology) and biotin-labeled anti-CD44 (IM7; BD Pharmingen) mAbs. Primary antibodies were detected with a Cy3-labeled anti-mouse Ab (Jackson ImmunoResearch Laboratories) and Alexa488-labeled avidin (Molecular Probes). For chemotaxis assays, T_N_ was pretreated with inhibitors for DOCK2 (CPYPP) and PI3Kγ (AS-605240; both Tocris) as indicated for 1 h at 37°C, and 0.5 x 10^6^ T_N_/well were added to Transwell chambers (5-µm pore size; Costar) to migrate to 50 nM CCL21 for 1.5 h at 37°C in the presence of the inhibitors. Cell viability was assessed by PI staining at the end of the experiment. Input and migrated T cells were enumerated by flow cytometry.

### Intravital Microscopy of Popliteal LN

(5-(and−6)-(((4-chloromethyl) benzoyl) amino) tetramethylrhodamine) (CMTMR, CellTracker orange) and Chloromethyl-coumarin (CMAC, CellTracker blue)-labeled WT, Itk^-/-^ or Tiam1^-/-^ CD4^+^ T_N_ (3 x 10^6^) were injected i.v. into sex-matched C57BL/6 mice 24–32 h prior to imaging. The right popliteal LN of recipient mice was surgically prepared as previously described (48). 2PM imaging was performed using a TrimScope 2PM system (LaVision Biotec) and a 20X objective (NA 0.95; Olympus). 16-slice z stacks with 4-µm spacing of 250 x 250 µm field of views were acquired every 20 s for 20 min. Imaging was performed in the T cell zone as identified by the presence of high endothelial venules (labeled with Alexa Fluor 633–coupled MECA-79; 10 µg/mouse). Volocity software (PerkinElmer) was used to generate volume-rendered 4D movies and for semiautomated tracking of cell motility. Mean single cell track speeds were calculated from the x,y,z coordinates of cell centroids using Matlab (The MathWorks) (49).

### Estimation of T Cell–DC Encounters

We simulated 200 8-h-long WT and Itk^-/-^ T cell tracks by sampling from control and Itk^-/-^ T cell tracks acquired by 2PM. As steps at higher velocities tend to have smaller turning angles, we drew combinations of speed, turning angle, and plane angle from the same step instead of drawing speed, turning angle, and plane angle separately. To additionally account for the correlation of velocity and turning angle, we repeatedly exchanged random steps of our samples, keeping only those exchanges that made the mean squared differences of consecutive speeds and turning angles of our synthetic track more similar to the same means of the measured tracks. We stopped exchanging when both means of the simulated track were smaller than those of the measured tracks. To simulate contact formation among a predefined number of synthetic T cells and a number of DCs, we randomly chose 100 simulated T cell tracks and moved them to starting positions normally distributed around the center of a spherical T cell zone of 1-mm diameter for 8 h as described (39). We chose the SD of 150 µm, such that half of the WT tracks resided within the T cell zone for an average half-life of 11 h (40). We considered all steps of the track within the T cell zone, including reentry as residency time. We then checked, step by step, the distance to 10’000 and 50 static DCs uniformly distributed throughout the T cell zone. We considered proximity ≥ 15 µm as a stable contact and did not allow T cells to move on. We repeated the different simulations 50 times to obtain stable distributions of outcomes. This analysis was performed using open Python libraries for scientific computing (https://github.com/germannp/lana/blob/master/lana/).

### *In Vivo* Homing and Homeostasis

WT, Itk^-/-^, or Tiam1^-/-^ T_N_ were labeled with Carboxyfluorescein succinimidyl ester (CFSE; Life Technologies) or eFluor 670 (e670; Life Technologies) for 15–20 min at 37°C in PBS, with dyes swapped between experiments, and 3 x 10^6^ cells of each were injected i.v. at a 1:1 ratio into 5- to 10-week-old C57BL/6J or MHCII^-/-^ recipient mice. At the indicated time points, blood and SLOs were isolated and analyzed by flow cytometry for percentage of transferred cells among CD4^+^ T cells. In WT recipients, we recovered between 0.22% (in PP) and 1.84% (in blood) of WT and Itk^-/-^ CD4^+^ T cells at 10 to 14 days post transfer, while in MHCII^-/-^ recipients, we recovered between 0.98% (in PP) and 8.37% (in blood) of transferred cells at 8 days post transfer.

### Statistical Analysis

Data were analyzed using Prism (GraphPad Software) using unpaired Student’s t-test or Mann–Whitney or Kruskal–Wallis tests. P-values < 0.05 were considered significant.

## Data Availability Statement

The original contributions presented in the study are included in the article/**Supplementary Material**. Further inquiries can be directed to the corresponding author.

## Ethics Statement

The animal study was reviewed and approved by Kanton of Bern and Fribourg Amt für Lebensmittelsicherheit und Veterinärwesen.

## Author Contributions

FT performed the experiments and analyzed the data with the help of SW. NR performed *in silico* analysis. JS designed the experiments, analyzed the data, and wrote the manuscript with input from all coauthors. All authors contributed to the article and approved the submitted version.

## Funding

This work was funded by Swiss National Foundation (SNF) project grant 31003A_172994 and 310030-200406 and Sinergia project CRSII5_170969 (to JS).

## Conflict of Interest

The authors declare that the research was conducted in the absence of any commercial or financial relationships that could be construed as a potential conflict of interest.

## Publisher’s Note

All claims expressed in this article are solely those of the authors and do not necessarily represent those of their affiliated organizations, or those of the publisher, the editors and the reviewers. Any product that may be evaluated in this article, or claim that may be made by its manufacturer, is not guaranteed or endorsed by the publisher.

## Supplementary Material

Supplementary Figure 1 CD44^high^ CD4^+^ T cell depletion and migration to CXCL12. **(A)** Representative flow cytometry plot showing CD44 and CD62L expression on WT and Itk^-/-^ CD4^+^ and CD8^+^ T cells. Numbers indicate percentage. **(B)** Representative flow cytometry plot of CD44 and CD62L expression on CD4^+^ T cells before and after negative isolation with CD44-coated beads. **(C)** Chemotaxis of WT and Itk^-/-^ CD4^+^ T_N_ treated with either CPYPP and/or AS-605204 towards 100 nM CXCL12. Graph shows percentage of transmigrated cells. Data in C were analyzed using an unpaired t-test and pooled from two independent experiments. *p < 0.05; ***p < 0.001.

Supplementary Figure 2 RNAseq expression level of Tec, Tiam and Vav family members in spleen CD4^+^ T_N_. Data are from Immgen database (www.immgen.org).
