# Supplementary figures and images for: The Tec Kinase Itk Integrates Naïve T Cell Migration and In Vivo Homeostasis
Source: Front Immunol. 2021 Sep 9;12:716405. doi: 10.3389/fimmu.2021.716405 (PMC8458560; doi:10.3389/fimmu.2021.716405)

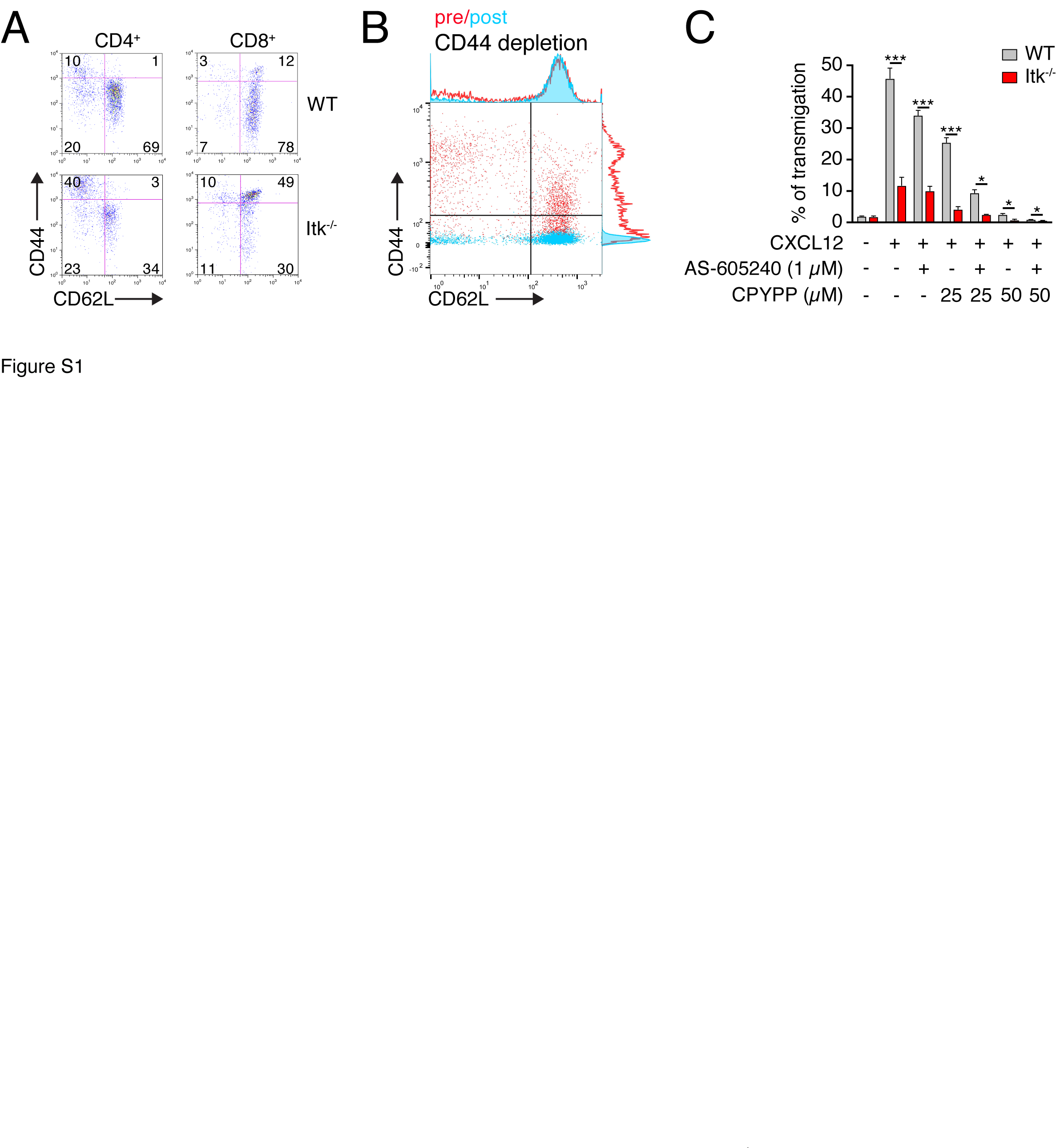

Supplement: Supplementary Figure 1 — CD44high CD4+ T cell depletion and migration to CXCL12. (A) Representative flow cytometry plot showing CD44 and CD62L expression on WT and Itk-/- CD4+ and CD8+ T cells. Numbers indicate percentage. (B) Representative flow cytometry plot of CD44 and CD62L expression on CD4+ T cells before and after negative isolation with CD44-coated beads. (C) Chemotaxis of WT and Itk-/- CD4+ TN treated with either CPYPP and/or AS-605204 towards 100 nM CXCL12. Graph shows percentage of transmigrated cells. Data in C were analyzed using an unpaired t-test and pooled from two independent experiments. *p < 0.05; ***p < 0.001. [file Image_1.tif]

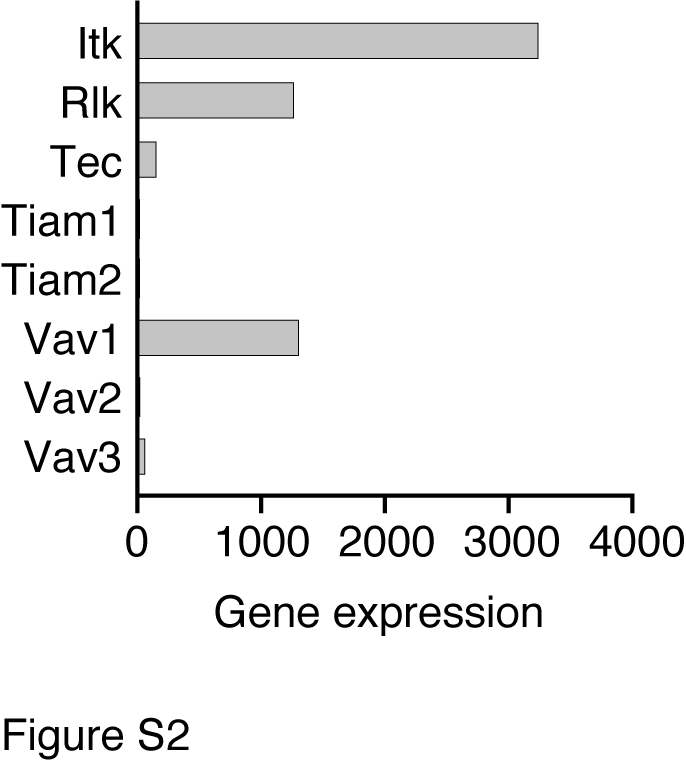

Supplement: Supplementary Figure 2 — RNAseq expression level of Tec, Tiam and Vav family members in spleen CD4+ TN. Data are from Immgen database (www.immgen.org). [file Image_2.tif]
